# Marital Status-Specific Associations Between Multidomain Leisure Activities and Cognitive Reserve in Clinically Unimpaired Older Adults: Based on a National Chinese Cohort

**DOI:** 10.3390/brainsci15040371

**Published:** 2025-04-03

**Authors:** Cheng Cai, Junyi Wang, Dan Liu, Jing Liu, Juan Zhou, Xiaochang Liu, Dan Song, Shiyue Li, Yuyang Cui, Qianqian Nie, Feifei Hu, Xinyan Xie, Guirong Cheng, Yan Zeng

**Affiliations:** 1 Hubei Provincial Clinical Research Center for Alzheimer’s Disease, Tianyou Hospital Affiliated to Wuhan University of Science and Technology, Wuhan University of Science and Technology, Wuhan 430065, China; cc19857169125@163.com (C.C.); wangjunyi0203@163.com (J.W.); liudan125@wust.edu.cn (D.L.); lj1527212@126.com (J.L.); 201702600@huat.edu.cn (J.Z.); liuxiaochang592@163.com (X.L.); 13035158128@163.com (D.S.); ssshiyue_li@163.com (S.L.); c18834563101@163.com (Y.C.); 17371093350@163.com (Q.N.); hufeifei@wust.edu.cn (F.H.); xiexinyan@wust.edu.cn (X.X.); chengguirong@wust.edu.cn (G.C.); 2 Brain Science and Advanced Technology Institute, Wuhan University of Science and Technology, Wuhan 430065, China

**Keywords:** leisure activities, marital status, community-dwelling older adults, cognitive function, longitudinal studies, Chinese Longitudinal Healthy Longevity Survey

## Abstract

**Background:** It is unclear how marital status moderates the association between multidomain leisure activities and the progression of cognitive decline in community-dwelling older adults. **Methods:** Data from the Chinese Longitudinal Healthy Longevity Survey with up to 10 years of follow-up were used. The study included participants aged ≥65 years without cognitive impairment at baseline. Cognitive function was assessed using the Mini-Mental State Examination (MMSE). Linear mixed-effect models were used to evaluate the modifying effect of marriage on leisure activities (multiple types, frequency, and single type) and cognitive decline. **Results:** A total of 5286 participants (aged 79.01 ± 9.54 years, 50.0% women, and 61.4% rural residents) were enrolled. The results indicated that marital status moderates the relationship between leisure activities and cognitive decline. In the unmarried group, multi-type and high-frequency leisure activities were more strongly associated with slower cognitive decline. Specific activities such as gardening, reading, performing household chores, and playing cards were found to significantly contribute to cognitive protection exclusively within the unmarried group, with no such effect observed in the married group. **Conclusions:** Marital status affects the relationship between participation in multiple leisure activities and cognitive decline in cognitively intact elderly people. For unmarried older adults, regular participation in leisure activities may be an effective intervention.

## 1. Introduction

Numerous epidemiological investigations [1,2] have suggested that high levels of engagement in leisure activities, particularly those that are cognitively stimulating [3,4], can delay cognitive decline [5,6] and alleviate the risk of dementia and Alzheimer’s disease (AD) [7]. The concept of cognitive reserve (CR) highlights the capacity of lifelong lifestyle choices and life experiences—measured by a combination of years of education, occupational complexity, and participation in hobbies and leisure activities [8]—to positively influence cognitive processes, enhancing efficiency and flexibility in coping with cognitive decline [9]. Accumulating evidence suggests that CR improves cognitive adaptability and reduces vulnerability to brain aging, pathology, or injury, thereby delaying the onset of clinical symptoms [9,10]. However, the influence of marital status as a social determinant on the relationship between leisure activities and cognitive reserve remains ambiguous. Marital status may affect an individual’s social support network, lifestyle choices, and mental health, thereby impacting both the extent of participation in leisure activities and cognitive functioning [11]. Current epidemiological research has yielded inconsistent results. For instance, a recent longitudinal study by Y. Lee et al. [12] based on a four-year follow-up from the Health and Retirement Study in the United States indicated that increased engagement in leisure activities could alleviate the adverse effects of widowhood on cognitive function among older adults, and the alleviating role was more pronounced in recently widowed individuals. Conversely, a short-term follow-up study conducted by Yura Lee et al. within Chinese immigrant populations revealed that leisure activities did not significantly moderate cognitive function among widowed individuals [13]. This discrepancy underscores the need for further investigation into how marital status influences the relationship between multidomain leisure activities and the progression of cognitive decline. Consequently, this study sought to explore the effects of marital status on the association between multidomain leisure activities and cognitive decline progression among community-dwelling older adults utilizing data from the Chinese Longitudinal Healthy Longevity Survey (CLHLS) [14]. The study aimed to provide insights that could inform the development of more targeted public health interventions, focusing on detailed comparisons across total amounts, varying frequency, and types of activities concerning cognitive decline.

## 2. Methods

### 2.1. Study Design and Participants

**Figure 1:**
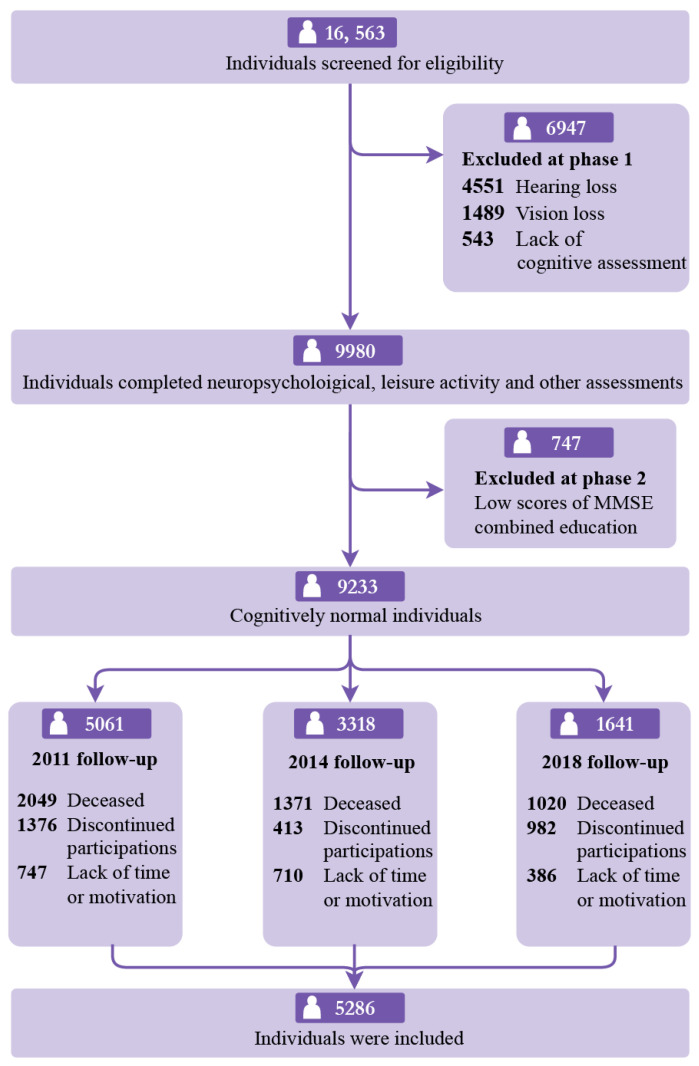
Study participants. MMSE = Mini-Mental State Examination.

The dataset was sourced from 2008 and 2018, following up on the CLHLS [14]. Participants aged ≥65 years who exhibited no signs of cognitive impairment at baseline were recruited for the study. These individuals had completed an initial survey during 2008–2009 and had participated in at least one follow-up survey conducted in the waves of 2011–2012, 2014, or 2017–2018. A total of 16,563 participants were initially extracted from the 2008 survey. We excluded 4551 individuals with severe hearing impairment, 1489 with severe visual impairment, 747 with cognitive impairment, and 543 without cognitive data at baseline. Additionally, we removed 4440 participants who passed away in 2011, 2014, and 2018 and 2771 individuals who were lost to follow-up. Ultimately, the study included 5286 participants. A flowchart detailing the sample selection process is presented in Figure 1. CLHLS cohort ethics approval was obtained from the Ethics Committee of Peking University (IRB00001052-13074), and all participants provided written informed consent.

### 2.2. Measurement of Multidomain Leisure Activity

The study defined leisure activities from the perspectives of productive activity and social attributes [15], ultimately including participants’ involvement in seven types of leisure activities: household chores, gardening, reading newspapers/books, raising domestic animals, playing cards and/or mahjong, watching television and/or listening to the radio, and participating in organized social activities.

Participants were requested to evaluate the amount and frequency with which they engaged in seven typical leisure activities for 30 min per time over the past 12 months. Participants responded on a 5-point Likert scale (never = 0, less than once a month = 1, less than once a week = 2, at least once a week = 3, almost every day = 4). The scores were aggregated to produce a total leisure activity figure that ranged from 0 to 28, with higher scores indicating greater involvement in leisure activities. Participation was subsequently classified into three frequency categories: low frequency (0–8 points), moderate frequency (9–12 points), and high frequency (13–28 points). Additionally, leisure activities were categorized into three levels of diversity based on the number of different activities engaged in: low diversity (0–1 type of activity), medium diversity (2–3 types of activities), and high diversity (4–7 types of activities).

### 2.3. Assessment of Marital Status

Self-reported marital status was classified into two categories: married, which encompassed married individuals either living or not cohabiting with their spouses, and unmarried, which included those who were divorced, widowed, or had never been married.

### 2.4. Assessment of Cognition

Participants completed the Chinese version of the Mini-Mental State Examination (MMSE), which evaluates eight dimensions of cognitive function: orientation, language, attention and calculation, visuospatial ability, immediate and delayed word recall, immediate and delayed logical memory, symbol digit modalities, and performance on the trail-making test [16]. The total MMSE score ranges from 0 to 30, with higher scores indicating better cognitive performance. Participants were identified as having cognitive impairment with MMSE scores < 17 for uneducated persons, <20 for persons with 1–6 years of education, and <24 for persons with more than 6 years of education [17].

### 2.5. Covariance

We considered the factors that might influence cognitive decline and leisure activity. These factors encompassed sociodemographic variables, including age, sex, educational attainment, residence, economic status, and pre-retirement occupation. Additionally, we considered lifestyle factors such as smoking, alcohol consumption, physical activity, dietary practices, and body mass index (BMI). Medical history was assessed through self-reported instances of hypertension, diabetes, heart disease, and stroke or cardiovascular conditions. Furthermore, we evaluated the presence of depression and disability. Educational attainment was quantified based on the self-reported duration of formal education received in early life. Participants’ self-assessments about their financial circumstances compared with their peers determined their economic status. Occupations were classified as white-collar, blue-collar, and agricultural as per the International Standard Classification of Occupations (ISCO) [18]. Smoking history, alcohol consumption, and exercise participation were categorized as no (never) or yes (previously or currently) (Table S1).

Dietary habits were assessed through self-reported food consumption frequency and classified as healthy and unhealthy dietary habits [19]. Table S2 presents a scoring system where one was assigned to each healthy eating habit and zero to unhealthy eating habits. The total scores were calculated by summing all items, with higher scores reflecting more nutritious eating behaviors. Disability was evaluated through basic activities of daily living (B-ADL) and instrumental activities of daily living (I-ADL). Depressive symptoms within the CLHLS were measured using a five-item scale that has been frequently utilized in various studies analyzing depressive symptoms within the CLHLS data [20].

### 2.6. Statistical Analyses

All data were analyzed using R software (version 4.4.1, The R Foundation for Statistical Computing, Vienna, Austria, https://www.r-project.org/, accessed on 14 June 2024). For normally distributed continuous variables, data are presented as means ± standard deviation (mean ± SD), and differences were compared using t-tests. For non-normally distributed continuous variables, data are presented as medians (Q1, Q3), and differences were compared using the non-parametric rank-sum test. Categorical variables are described using proportions, and differences were compared using the χ^2^ tests.

In this study, a total of 5286 samples were included, with the highest missing data proportion being 2.2% (Figure S1). Given the extremely low missing data rate (<3%) and in accordance with methodological guidelines [21,22], we adopted complete case analysis as the primary strategy.

We used linear mixed models to examine the interactive effects of leisure activity (multiple types, frequency, and single type), marital status, and time (years from baseline) on longitudinal cognition. After observing the interaction effect of marital status on leisure activities and cognitive decline, stratified analyses were conducted by marital status. All models included random intercepts and slopes. We also performed sensitivity analyses to examine the robustness of the findings, including repeating the main analysis with (1) exclusion of participants with shorter follow-up (years < 4) to avoid potential differential bias, (2) restriction to participants with two or more follow-up MMSE assessments, (3) to reduce the potential impact of social isolation on the results, the question “Do you often feel lonely and isolated?” in the depression scale was added to the model as an independent covariate, and (4) the heterogeneity of effects was further tested by stratification by age group (65–74 and ≥75) and gender. A two-sided test was used with a significance level of α = 0.05.

## 3. Results

### 3.1. Baseline Characteristics of Study Samples

Table 1 summarizes the baseline characteristics of the participants. In sum, 5286 (aged 79.01 ± 9.54 years, educated 2.82 ± 3.77 years, 50.0% women, and 61.4% rural residents) had completed two to four follow-up evaluations. The baseline MMSE scores ranged from 17 to 30 (mean = 27.23, standard deviation [SD] = 3.05). Statistically significant differences were observed between married and unmarried groups concerning age, sex, years of education, economic status, occupation, exercise, smoking, alcohol consumption, diet, depression status, BMI, diabetes, heart disease, stroke, ADL, MMSE scores, leisure scores, frequency, and diversity (*p* < 0.001).

### 3.2. Marital Status-Specific Associations of Leisure Activity Diversity with Cognitive Decline

**Figure 2:**
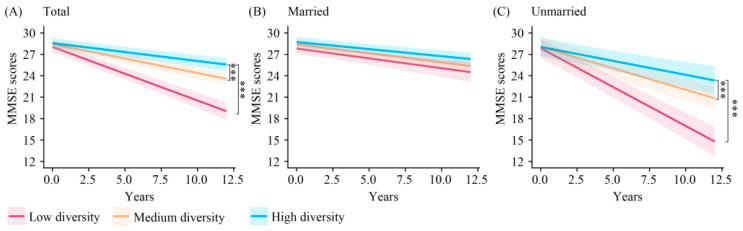
Longitudinal change in cognition among low-, medium-, and high-diversity leisure activity groups in different marital populations. *** *p* < 0.001. MMSE: Mini-Mental State Examination.

This research further investigated marital status-specific associations of leisure activity diversity, specifically the number of leisure activities in which participants engaged with cognitive decline. In models that were fully adjusted, participants who engaged in high- (*β* = 0.500, 95% CI: 0.396–0.603)- and medium-diversity activities (*β* = 0.347, 95% CI: 0.250–0.444) at baseline demonstrated a slower rate of cognitive decline in comparison to those with low diversity (Figure 2A and Table 2). Significant interactions were observed between marital status, leisure activity diversity, and time (*p* < 0.001). In subsequent stratified analyses, the unmarried group, but not the married group, high- (*β* = 0.697, 95% CI: 0.495–0.900) and medium-diversity activities (*β* = 0.489, 95% CI: 0.319–0.659) were associated with a slower rate of cognitive decline (Figure 2B,C, and Table 2) than low-diversity activities.

### 3.3. Marital Status-Specific Association of Leisure Activity Amount and Frequency with Cognitive Decline

Table S3 shows marital status-specific associations of leisure activity amount and frequency with cognitive progression. In a fully adjusted model, an increase of one point in the leisure activity amount was associated with a reduction of 0.029 points in cognitive decline (95% confidence interval [CI]: 0.023–0.036). Significant interactions among marital status, leisure activity amount, and time were related to cognitive decline (*p* < 0.001). In the stratified analysis based on marital status, unmarried participants exhibited a more pronounced association between higher leisure activity and a slower rate of cognitive decline (*β* = 0.042, 95% CI: 0.028–0.056), while the married group demonstrated only a marginally slower decline (*β* = 0.009, 95% CI: 0.002–0.016).

This research additionally examined the annual rates of cognitive decline among participants categorized by varying activity frequency. In comparison to the low-frequency group, both the moderate-frequency (*β* = 0.174, 95% CI: 0.103–0.246) and the high-frequency groups (*β* = 0.286, 95% CI: 0.208–0.363) were associated with slower rates of cognitive decline (Table S4). Significant interactions were observed among marital status, high-frequency leisure activities, and time (*p* < 0.001). In the stratified analysis by marital status, in the unmarried group, high-frequency leisure activity was associated with a slower rate of cognitive decline (*β* = 0.413, 95% CI: 0.232–0.593) compared to low-frequency leisure activity. However, the married group showed only a marginally reduced rate of cognitive decline (*β* = 0.099, 95% CI: 0.022–0.176).

### 3.4. Marital Status-Specific Association of Individual Activity Types with Cognitive Decline

**Figure 3:**
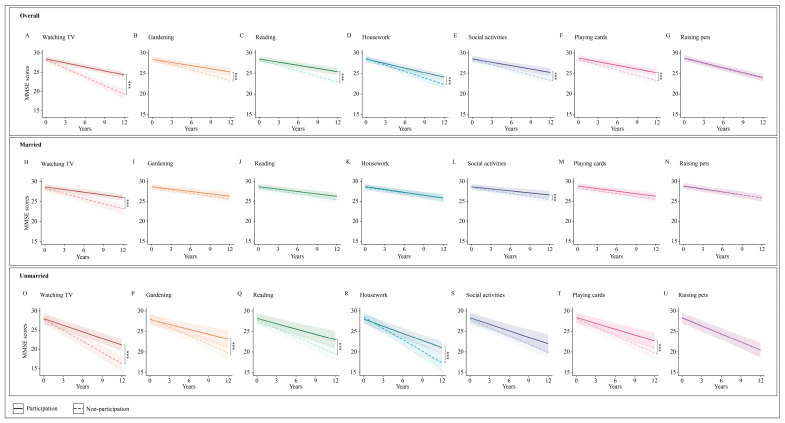
Marital status-specific longitudinal changes in MMSE scores over 10 years among participants engaged in different leisure activities. *** *p* < 0.001. MMSE = Mini-Mental State Examination.

Figure 3 and Table S5 show marital status-specific associations of individual activity types with cognitive decline. In a fully adjusted model, compared to no leisure activities, watching TV (β = 0.356, 95% CI: 0.124–0.475), gardening (β = 0.299, 95% CI: 0.124–0.475), reading (β = 0.140, 95% CI: 0.084–0.195), household chores (β = 0.140, 95% CI: 0.075–0.204), playing cards (β = 0.103, 95% CI: 0.048–0.159), and social activities (β = 0.102, 95% CI: 0.039–0.165) were associated with a reduced rate of cognitive decline, whereas keeping pets did not significantly lower the rate of cognitive decline (0.005, 95% CI: −0.045–0.055).

Significant interactions were found among marital status, time, and four types of activity: gardening, reading, household chores, and playing cards. Stratified analysis based on marital status indicated that within the unmarried group, but not in the married group, engagement in gardening (*β* = 0.299, 95% CI: 0.124–0.475), reading (*β* = 0.261, 95% CI: 0.086–0.436), household chores (*β* = 0.345, 95% CI: 0.186–0.504), and playing cards (*β* = 0.219, 95% CI: 0.059–0.380) were linked to a reduced rate of cognitive decline.

### 3.5. Sensitivity Analyses

To verify the robustness of the core conclusions, we performed multidimensional sensitivity analysis. First, after excluding participants with a follow-up time of <4 years or limiting the sample to those who completed ≥2 MMSE assessments, the effect size of the unmarried group remained stable, supporting the reliability of the results (Tables S6 and S7). Second, subjective social isolation (measured by the question “Do you often feel lonely and isolated?”) was included in the model as an independent covariate. The results showed that the cognitive protective effect of high-diversity activities in the unmarried group remained robust (Table S8). Further stratified analysis found that the participation of elderly unmarried people (≥75 years old) in leisure activities was more strongly associated with cognitive building. In addition, housework and reading had significant effects on elderly or male unmarried groups, and gardening had a significant effect on female unmarried people (Tables S9 and S10).

## 4. Discussion

In a large nationally representative cohort of older Chinese adults initially free from dementia, our findings indicate that increased engagement in diverse and stimulating leisure activities correlates with a subsequent decrease in cognitive decline over an average follow-up period of 5.67 years. This association remains significant after controlling for demographic variables, socioeconomic status, lifestyle choices, dietary habits, and health conditions. Notably, the cognitive protective benefits of leisure activities were particularly evident among individuals who were unmarried, including those who were divorced, separated, widowed, or never married. In the married group, only leisure activities characterized by high frequency were linked to a reduced rate of cognitive decline, while low- and moderately frequent activities did not exhibit a similar association. Activities such as gardening, reading, performing household chores, and playing cards were found to significantly contribute to cognitive protection exclusively within the unmarried group, with no such effect observed in the married group. This research underscores the marital status-specific relationship between leisure activities and cognitive reserve and highlights the importance of encouraging unmarried individuals to pursue diverse leisure activities in later life.

This study focused on unmarried people’s participation in diverse leisure activities in maintaining cognitive function in their later years. Compared with married people, older unmarried people may benefit more from leisure activities, particularly gardening, reading, housework, and playing cards. These findings align with previous studies concerning widowed individuals, who often mitigate the negative impacts of spousal loss through participation in mentally stimulating activities to protect cognitive functions [23]. In particular, participating in cognitive activities, such as playing mahjong and reading, are believed to contribute to the delay or prevention of dementia [24]. The underlying mechanisms through which unmarried individuals derive more significant advantages from leisure activities remain ambiguous, as does the potential relationship between leisure activities and cognitive decline. An increasing quantity of research indicates that stressful life events, especially those that jeopardize intimate relationships, are associated with a heightened risk of dementia [25]. The loss of a spouse or the experience of divorce represents a significant source of stress in an individual’s life, resulting in profound psychological and physical distress. Furthermore, married people’s cognitive stimulation mainly comes from daily interactions with their spouses [26], which provide stable cognitive input and maintain cognitive function through continuous social exchanges. However, unmarried people lack such intimate partner relationships and need to actively establish alternative social networks through leisure activities [27]. Such experiences are negatively correlated with cognitive outcomes [28], while participating in leisure activities (voluntary and pleasant non-work activities, such as hobbies, arts, voluntary activities, community groups, sports, and social activities) enhances interpersonal relationships and social connections, thereby fostering psychological resilience and improving coping strategies. Consequently, unmarried individuals may be able to manage neuropathological challenges through compensatory mechanisms [29] more effectively. Finally, individual personality traits may play an important role in the relationship between marital status and cognitive decline [30,31], with marriage itself perhaps not directly causing cognitive differences. Individuals with higher levels of neuroticism tend to avoid social activities, thus reducing exposure to cognitive stimulation [30].

Our research indicates that the cognitive decline experienced by married older adults occurs more slowly than that of their unmarried counterparts. This finding suggests that the married population has obtained cognitive health benefits from communication with their spouses, akin to the role offered by leisure activities. Furthermore, having a marital partner influences individuals’ capacity to make healthier lifestyle choices. Spouses can be pivotal in promoting healthy habits, monitoring their partner’s health, and providing essential social support [32]. While married individuals experience cognitive health benefits from their unions, it is evident that additional cognitive health advantages can be gained through increased participation in leisure activities. Therefore, married older adults need to participate in leisure activities more frequently (more than four times a week) to maximize these health benefits, as our results show. Conversely, unmarried older adults may derive greater health benefits from leisure activities than their married peers. Although the effect size is medium, its clinical significance may lie in the long-term cumulative effect of slowing cognitive decline. Previous studies have shown that the rate of cognitive decline is closely related to health outcomes in older adults. For example, a study by Lv et al. [33] found that for every 1 point/year increase in cognitive decline, the risk of death increased by 11%. Therefore, even a small delay in cognitive decline may have a substantial impact on an individual’s health outcomes after long-term follow-up. This highlights the importance of promoting leisure activities as a potential public health strategy to mitigate cognitive decline, especially among vulnerable populations such as unmarried older adults.

This study did not consider changes in marital status. However, changes in marital status, especially the loss of a spouse, have a profound impact on cognitive function and overall health in the elderly. The death of a spouse may not only increase psychological stress but also weaken the social support network, both of which have a negative impact on cognitive health [34]. In addition, the death of a spouse may have different effects on mental [35] and physical health [36,37,38], and widowhood often causes more lasting damage to physical health [35,36,37,38]. Therefore, future studies should focus on the impact of changes in marital status on cognitive function and explore the role of social support in this process and how to alleviate the negative impact of the loss of a spouse through intervention measures to promote cognitive health and quality of life in the elderly.

Social isolation, a core social and psychological factor affecting the cognitive health of unmarried elderly people, forms a dual mechanism of action by directly accelerating neurodegenerative lesions and indirectly regulating the protective effect of leisure activities: on the one hand, unmarried people are more likely to fall into social isolation due to lack of spouse support, which damages hippocampal neuroplasticity by upregulating inflammatory factors and chronic stress [39], leading to cognitive decline; on the other hand, leisure activities partially offset the harm of isolation by enhancing social connection and psychological resource compensation [40]. Sensitivity analysis shows that after controlling for subjective social isolation, the modifying effect of marriage on the association between leisure activities and cognitive decline remains robust, suggesting that its protective mechanism may be partially independent of social connection. In addition, the moderating effect shows significant gender heterogeneity: unmarried women are more likely to achieve stronger cognitive protection through gardening and reading clubs, while unmarried men are more likely to achieve stronger cognitive protection through housework, but as the frequency and type of leisure activities increase, the gender difference becomes less obvious, which may be related to the different leisure activity patterns between men and women in old age [41,42]. Unmarried women may view housework as a personal choice rather than a familial obligation [43,44]. In the future, it is necessary to further explore the impact of different leisure patterns of unmarried men and women on cognitive decline.

The leisure activities used in this study were assessed through self-report, which may be affected by recall bias. Older people, especially those with cognitive impairment or memory loss, may underestimate the frequency and duration of their activities [45,46]. However, excluding participants with cognitive impairment at baseline can help to mitigate recall bias to a certain extent and reduce the underestimation of activity frequency by those with early cognitive impairment due to memory impairment. In addition, the sensitivity analysis results showed that the effect size was lower in those aged 65–74 years, which may be because the overall cognitive reserve of younger elderly people is higher and the buffering effect of activities on decline has not yet become prominent [47,48].

This research has several strengths: (1) the data were derived from a large-scale, nationally representative aging cohort in China, which has been subjected to long-term follow-up, thereby enhancing the reliability of the findings; (2) the incorporation of comprehensive indicators of leisure activity diversity, interaction effects, and hierarchical analysis facilitated a critical assessment of the cognitive protective effects of multidomain leisure activities among unmarried individuals; and (3) a series of sensitivity analyses and adjustments for various confounding factors contribute to the robustness of the results. Nevertheless, this study is not without limitations. First, leisure activities were assessed through self-report, which may be subject to recall bias. Older adults, particularly those with cognitive impairment or memory decline, may struggle to accurately recall the frequency and duration of their activities, potentially compromising data accuracy. Second, cognitive function was assessed solely using the MMSE, which lacks sensitivity to early or subtle cognitive decline, provides limited information on specific cognitive domains, and is susceptible to educational and cultural biases (e.g., lower scores in individuals with limited education). Although education-adjusted thresholds were applied, this assessment may still fail to fully capture subtle changes in cognitive function. Moreover, this study categorized divorced, widowed, and never-married individuals into a single “unmarried” group to enhance statistical power. While necessary due to the small sample of divorced and never-married participants, this approach may obscure critical social, psychological, and behavioral differences among subgroups. For instance, widowed individuals may experience social support disruption, whereas lifelong never-married individuals may have adapted to independent living. Consequently, the observed associations in the unmarried group may primarily reflect the characteristics of the widowed population. Additionally, the exclusion of participants who died or were lost to follow-up may have introduced survivor bias, leading to a sample skewed toward relatively healthier older adults, which could have resulted in an overestimation of the cognitive benefits of leisure activities. The exclusion of individuals with sensory impairments may also underestimate the bidirectional relationship between sensory impairments, cognition, and leisure activity [49]. Similarly, excluding individuals with early neurodegenerative diseases may lead to an overestimation of the cognitive benefits of leisure activities in “healthy older adults,” making generalization to high-risk populations less reliable. Furthermore, this study only considered the association between baseline marital status, leisure activity engagement, and cognitive function, without accounting for changes in marital status and leisure activity over time. If the decline in activity over time coincides with accelerated cognitive decline, it may confound the true association between leisure activity and cognition. Additionally, if individuals with low baseline activity levels increase their activity later, their cognitive decline may be slowed, potentially leading to an underestimation of the effect. Finally, given the observational design of the study, the current results only reveal an association between leisure activity and cognitive function, and a clear causal relationship cannot be established.

## 5. Conclusions

In conclusion, this research highlights the significant association between increased frequency and variety of leisure activities in later life and enhanced cognitive functioning. It suggests that leisure activities are more helpful in protecting the cognitive function of unmarried older individuals. Since all leisure activities included in this study are adjustable lifestyle behaviors, encouraging the elderly to participate in diversified leisure activities may be a low-cost and feasible intervention for cognitive decline. Future investigations should address the existing limitations to furnish evidence-based recommendations for preventing cognitive deterioration.

## Figures and Tables

**Table 1:** Baseline characteristics based on marital status (*n* = 5286).

Characteristic	Overall (*n* = 5286)	Married (*n* = 2809)	Unmarried (*n* = 2477)	F/χ^2^	*p* Value
Age (years), Mean ± SD	79.01 ± 3.77	75.03 ± 7.62	83.53 ± 9.49	127.81	<0.001
Sex, *n* (%)				511.92	<0.001
Male	2645 (50.0%)	1816 (64.6%)	829 (33.5%)		
Female	2641 (50.0%)	993 (35.4%)	1648 (66.5%)		
Education (years), Mean ± SD	2.82 ± 3.77	3.73 ± 4.05	1.80 ± 3.14	257.23	<0.001
Residence, *n* (%)				2.451	0.294
Country	3248 (61.4%)	1706 (60.7%)	1542 (62.3%)		
Town	1107 (20.9%)	587 (20.9%)	520 (21.0%)		
City	931 (17.6%)	516 (18.4%)	415 (16.8%)		
Economic status, *n* (%)				12.36	0.002
Poor	753 (14.3%)	414 (14.8%)	339 (13.7%)		
Average	3697 (70.0%)	1996 (71.2%)	1701 (68.7%)		
Rich	830 (15.7%)	395 (14.1%)	435 (17.6%)		
Occupation, *n* (%)				82.237	<0.001
White-collar	538 (10.4%)	382 (13.9%)	156 (6.4%)		
Blue-collar	1097 (21.2%)	592 (21.6%)	505 (20.8%)		
Farmer	3534 (68.4%)	1771 (64.5%)	1763 (72.7%)		
Exercise (Yes), *n* (%)	1987 (37.6%)	1138 (40.5%)	849 (34.3%)	21.83	<0.001
Smoking (Yes), *n* (%)	2055 (38.9%)	1348 (48.0%)	1461 (52.0%)	208.85	<0.001
Drinking (Yes), *n* (%)	1853 (35.1%)	1155 (41.1%)	698 (28.2%)	96.79	<0.001
Dietary habit, Mean ± SD	3.26 ± 1.54	3.40 ± 1.56	3.11 ± 1.51	11.07	<0.001
Depression, Mean ± SD	6.53 ± 3.13	6.02 ± 2.91	7.10 ± 3.26	37.17	<0.001
Hypertension (Yes), *n* (%)	1145 (22.1%)	622 (22.5%)	523 (21.5%)	0.774	0.379
Diabetes (Yes), *n* (%)	160 (3.1%)	110 (4.0%)	50 (2.1%)	15.87	<0.001
Heart disease (Yes), *n* (%)	503 (9.7%)	293 (10.6%)	210 (8.6%)	5.61	0.018
Stroke (Yes), *n* (%)	258 (4.9%)	159 (5.7%)	99 (4.1%)	7.69	0.006
ADL, Mean ± SD	15.92 ± 3.53	15.12 ± 2.72	16.82 ± 4.089	428.94	<0.001
BMI (kg/m^2^), Mean ± SD	21.17 ± 3.57	21.63 ± 3.62	20.63 ± 3.43	1.179	<0.001
MMSE score, Mean ± SD	27.2 ± 3.05	27.91 ± 2.53	26.47 ± 3.38	333.65	<0.001
Leisure activity score, Mean ± SD	9.75 ± 4.74	10.79 ± 4.65	8.57 ± 4.55	47.57	<0.001
Leisure activity frequency, *n* (%)				240.84	<0.001
T1	2409 (45.6%)	1034 (36.8%)	1375 (55.5%)		
T2	1665 (31.5%)	929 (33.1%)	736 (29.7%)		
T3	1212 (22.9%)	846 (30.1%)	366 (14.8%)		
Leisure activity variety, *n* (%)				292.05	<0.001
0–1	809 (15.3%)	268 (9.5%)	514 (21.8%)		
2–3	2923 (55.3%)	1472 (52.4%)	1451 (58.6%)		
4–7	1554 (29.4%)	1069 (38.1%)	485 (19.6%)		

*Notes:* ADL = activities of daily living, BMI = body mass index, MMSE = Mini-Mental State Examination.

**Table 2:** Marital status-specific leisure activity variety and cognitive decline.

Variables	Model 1 *		Model 2 ^†^	
	*β* (95% *CI*)	*p* Value	*β* (95% *CI*)	*p* Value
Overall population:				
(0–1) × time	Reference		Reference	
(2–3) × time	**0.365 (0.272–0.459)**	**<0.001**	**0.347 (0.250–0.444)**	**<0.001**
(4–7) × time	**0.511 (0.411–0.610)**	**<0.001**	**0.500 (0.396–0.603)**	**<0.001**
Three-way interaction:				
(0–1 × time) × marital status	Reference		Reference	
(2–3 × time) × marital status	**0.426 (0.236–0.617)**	**<0.001**	**0.437 (0.240–0.633)**	**<0.001**
(4–7 × time) × marital status	**0.562 (0.355–0.769)**	**<0.001**	**0.578 (0.364–0.792)**	**<0.001**
Marital status-stratified two-way interactions:				
(0–1) × time married	Reference		Reference	
(2–3) × time married	0.035 (−0.080–0.150)	0.552	0.021 (−0.098–0.140)	0.733
(4–7) × time married	0.085 (−0.032–0.203)	0.156	0.077 (−0.045–0.198)	0.216
(0–1) × time unmarried	Reference		Reference	
(2–3) × time unmarried	**0.487 (0.337–0.638)**	**<0.001**	**0.489 (0.319–0.659)**	**<0.001**
(4–7) × time unmarried	**0.684 (0.505–0.863)**	**<0.001**	**0.697 (0.495–0.900)**	**<0.001**

*Notes*: * Model 1 was the demographically adjusted model. Demographic adjustment included sex, age, education, residence, economic status, and occupation. ^†^ Model 2 was the full covariate-adjusted model. The full covariate adjustment additionally accounted for exercise, smoking, drinking, diet habits, depression, hypertension, diabetes, stroke/cardiovascular disease, ADL, and BMI. Bold indicates significant values.

## Data Availability

The datasets used in this article are publicly available from the CLHLS. The questionnaires and datasets are free to download at https://opendata.pku.edu.cn/dataset.xhtml?persistentId=doi:10.18170/DVN/WBO7LK, accessed on 14 June 2024.

## Supplementary Materials

The following supporting information can be downloaded at: https://www.mdpi.com/article/10.3390/brainsci15040371/s1, Table S1. The definition of covariates; Table S2. The definition of dietary habits; Table S3. Marital status-specific leisure activity scores and cognitive decline; Table S4. Marital status-specific leisure activity frequency and cognitive decline; Table S5. Marital status-specific individual leisure activity and cognitive decline *; Table S6. Sensitivity analysis results after excluding short follow-up periods; Table S7. Sensitivity analysis results after restriction to participants with two or more follow-up MMSE assessments; Table S8. Sensitivity analysis results after considering subjective social isolation as an independent covariate; Table S9. Age and marital status-specific leisure activity and cognitive decline; Table S10. Sex and marital status-specific leisure activity and cognitive decline; Figure S1. Proportion of missing data at baseline for covariates.
