# Supplementary material for: Marital Status-Specific Associations Between Multidomain Leisure Activities and Cognitive Reserve in Clinically Unimpaired Older Adults: Based on a National Chinese Cohort
Source: Brain Sci. 2025 Apr 3;15(4):371. doi: 10.3390/brainsci15040371 (PMC12025604; doi:10.3390/brainsci15040371)
Supplement: Supplementary file 1 [file brainsci-15-00371-s001.zip › brainsci-3514033-supplementary.pdf]

SUPPLEMENTARY MATERIAL

Marital-specific associations between multi-domain leisure activities and cognitive function in clinically unimpaired older adults: based on a national Chinese cohort

Cheng Cai [1,2,†], Junyi Wang [1,2,†], Dan Liu [1,2], Jing Liu [1,2], Juan Zhou [1,2], Xiaochang Liu [1,2], Dan Song [1,2], Shiyue Li [1,2], Yuyang Cui [1,2], Qianqian Nie [1,2], Feifei Hu [1,2], Xinyan Xie [1,2], Guirong Cheng [1,2] and Yan Zeng [1,2,*]

[1] Hubei Provincial Clinical Research Center for Alzheimer's Disease, Tianyou Hospital Affiliated to Wuhan University of Science and Technology, Wuhan University of Science and Technology, Wuhan 430065, China; cc19857169125@163.com (C.C.); wangjunyi0203@163.com (J.W.); liudan125@wust.edu.cn (D.L.); lj1527212@126.com (J.L.); 201702600@huat.edu.cn (J.Z.); liuxiaochang592@163.com (X.L.); 13035158128@163.com (D.S.); ssshiyue_li@163.com (S.L.); c18834563101@163.com (Y.C.); 17371093350@163.com (Q.N.); hufeifei@wust.edu.cn (F.H.); xiexinyan@wust.edu.cn (X.X.); chengguirong@wust.edu.cn (G.C.)
[2] Brain Science and Advanced Technology Institute, Wuhan University of Science and Technology, Wuhan 430065, China

* Correspondence: zengyan68@wust.edu.cn
† These authors contributed equally to this work.

Contents

Table S1. The definition of covariates ... 2
Table S2. The definition of dietary habits ... 4
Table S3. Marital status-specific leisure activity scores and cognitive decline ... 5
Table S4. Marital status-specific leisure activity frequency and cognitive decline ... 6
Table S5. Marital status-specific individual leisure activity and cognitive decline* ... 7
Table S6. Sensitivity analysis results after excluding short follow-up periods ... 8
Table S7. Sensitivity analysis results after restriction to participants with two or more follow-up MMSE assessments ... 9
Table S8. Sensitivity analysis results after considering subjective social isolation as an independent covariate ... 10
Table S9. Age and marital status-specific leisure activity and cognitive decline ... 11
Table S10. Sex and marital status-specific leisure activity and cognitive decline ... 12
Figure S1. Proportion of missing data at baseline for covariates ... 13

Table S1. The definition of covariates

Category	Covariates	According to baseline self-report, or ID card	Coding of variables
Sociodemographic factors	Age	The age registered on the ID card	Continuous variable
	Sex	Biological sex	Male; female
	Residence	Actual residence over 10 years	Rural; town; urban
	Education	Years of schooling	Continuous variable
	Economic status	Participants' self-evaluations of the economic status of those around them	Poor; average; rich
	Occupation before retirement	Participants self-reported according to the International Standard Classification of Occupations (ISCO)	White-collar (governmental, institutional, or managerial personnel; professional and technical personnel; or military personnel), blue-collar (commercial service, industrial worker, self-employed, or houseworker), and farmers
Lifestyle factors	Smoking	Smoking history (previously or currently smoking = yes, never smoking = no)	Yes; No
	Drinking	Consuming alcohol (previously or currently drinking = yes, never drinking = no)	Yes; No
	Regular physical exercise	Regularly doing exercise at present? (previously or currently = yes, never = no)	Yes; No
	Body mass index	BMI was calculated based on measured weight and height (weight in kilograms [kg] divided by squared height in centimeters) at baseline	Continuous variable
Medical history	Hypertension	Self-reported diagnosis	Yes; No
	Diabetes	Self-reported diagnosis	Yes; No

	Heart disease	Self-reported diagnosis	Yes; No
	Stroke	Self-reported diagnosis	Yes; No
Psychology	Depression	Five items have been often adopted to indicate depressive symptoms. (1) “Do you look on the bright side?”; (2) “Are you as happy now as when you were young?”; (3) “Do you often feel anxious or fearful?”; (4) “Do you often feel lonely and isolated?”; (5) “Do you feel the older you get the more useless you are?”	Continuous variable. A score from 0 to 4 was assigned to each response, with a higher score indicating a higher frequency of feeling negative.
Function	Disability	Basic activities of daily living (B-ADL) and instrumental activities of daily living (I-ADL)	Continuous variable

Table S2. The definition of dietary habits

	Healthy eating habits (1 score)	Unhealthy food habits (0 score)
Staple food	Whole grains	Non-whole grains
Main cooking oil	Vegetable oil	Animal oil
Eating fruit and vegetables, eggs, beans, dairy products, and nuts	Almost every day	No daily
Eating fish	At least once a week	Less than once a week
Eating sugar and desserts	Less than once a month	At least once a month

Table S3. Marital status-specific leisure activity scores and cognitive decline

Variables	Model 1*		Model 2†	
	β (95% *CI*)	*p* value	β (95% *CI*)	*p* value
Overall population:				
Leisure activity score × time	0.030 (0.024 – 0.036)	<0.001	0.029 (0.023 – 0.036)	<0.001
Three-way interaction:				
(Leisure activity score × time)×marital status	0.030 (0.017 – 0.043)	<0.001	0.030 (0.017 – 0.044)	<0.001
Marital status -stratified two-way interactions:				
Leisure activity score × time in the married	0.009 (0.002 – 0.016)	0.007	0.009 (0.002 – 0.016)	0.011
Leisure activity score × time in the unmarried	0.042 (0.030 – 0.055)	<0.001	0.042 (0.028 – 0.056)	<0.001

Notes: ADL = activity of daily living, BMI = body mass index.

* Model 1 was the demographically adjusted model. Demographic adjustment includes sex, age, education, residence, economic status, and occupation.

† Model 2 was the full covariate-adjusted model. The full covariate adjustment additionally accounts for exercise, smoking, drinking, diet habits, depression, hypertension, diabetes, stroke/cardiovascular disease, ADL, and BMI.

Table S4. Marital status-specific leisure activity frequency and cognitive decline

Variables	Model 1*		Model 2†	
	β (95% *CI*)	*p* value	*β* (95% *CI*)	*p* value
Overall population:				
T1 × time	Reference		Reference	
T2 × time	0.186 (0.117–0.254)	<0.001	0.174 (0.103 – 0.246)	<0.001
T3 × time	0.286 (0.211–0.361)	<0.001	0.286 (0.208 – 0.363)	<0.001
Three-way interaction:				
(T1 × time) ×marital status	Reference		Reference	
(T2 × time) ×marital status	0.100 (-0.037 – 0.238)	0.153	0.097 (-0.046 – 0.240)	0.183
(T3 × time) ×marital status	0.266 (0.108 – 0.423)	0.001	0.281 (0.118 – 0.444)	0.001
Marital status -stratified two-way interactions:				
T1 × time in the married	Reference		Reference	
T2 × time in the married	0.089 (0.018 – 0.161)	0.014	0.084 (0.009 – 0.158)	0.028
T3 × time in the married	0.099 (0.026 – 0.173)	0.008	0.099 (0.022 – 0.176)	0.011
T1 × time in the unmarried	Reference		Reference	
T2 × time in the unmarried	0.200 (0.072 – 0.329)	0.002	0.190 (0.044 – 0.336)	0.011
T3 × time in the unmarried	0.395 (0.235 – 0.555)	<0.001	0.413 (0.232 – 0.593)	<0.001

Notes: ADL = activity of daily living, BMI = body mass index.

* Model 1 was the demographically adjusted model. Demographic adjustment includes sex, age, education, residence, economic status, and occupation.

† Model 2 was the full covariate-adjusted model. The full covariate adjustment additionally accounts for exercise, smoking, drinking, diet habits, depression, hypertension, diabetes, stroke/cardiovascular disease, ADL, and BMI.

Table S5. Marital status-specific individual leisure activity and cognitive decline*

Variables	Overall β (95% *CI*)	Married β (95% *CI*)	Unmarried β (95% *CI*)	*P* for interaction
Watching TV × time	0.356 (0.279 – 0.433)	0.207 (0.087 – 0.326)	0.388 (0.226 – 0.550)	0.112
Gardening × time	0.145 (0.086 – 0.205)	0.053 (-0.019 – 0.126)	0.299 (0.124 – 0.475)	0.005
Reading × time	0.140 (0.084 – 0.195)	0.051 (-0.015 – 0.117)	0.261 (0.086 – 0.436)	0.020
Housework × time	0.140 (0.075 – 0.204)	-0.010 (-0.093 – 0.074)	0.345 (0.186 – 0.504)	<0.001
Social activities × time	0.102 (0.039 – 0.165)	0.085 (0.010 – 0.160)	0.142 (-0.046 – 0.330)	0.637
Playing cards × time	0.103 (0.048 – 0.159)	0.030 (-0.040 – 0.101)	0.219 (0.059 – 0.380)	0.020
Raising pets × time	0.005 (-0.045 – 0.055)	-0.024 (-0.087 – 0.039)	-0.007 (-0.143 – 0.128)	0.747

Notes: ADL = activity of daily living, BMI = body mass index.

* A full covariate-adjusted model was used. Full covariates adjustment included sex, age, education, residence, economic status, occupation, exercise, smoking, drinking, diet habit, depression, hypertension, diabetes, stroke/cardiovascular disease, ADL, and BMI.

Table S6. Sensitivity analysis results after excluding short follow-up periods

Variables	Overall<br>*β* (95% *CI*)	Married<br>*β* (95% *CI*)	Unmarried<br>*β* (95% *CI*)	*P* for interaction
Watching TV × time	0.307 (0.218 – 0.395)	0.197 (0.075 – 0.320)	0.283 (0.147 – 0.418)	0.367
Gardening × time	0.130 (0.061 – 0.199)	0.045 (-0.027 – 0.117)	0.225 (0.086 – 0.364)	0.016
Reading × time	0.115 (0.052 – 0.179)	0.042 (-0.025 – 0.108)	0.133 (-0.008 – 0.273)	0.220
Housework × time	0.049 (-0.028 – 0.126)	-0.050 (-0.135 – 0.034)	0.183 (0.043 – 0.324)	0.003
Social activities × time	0.097 (0.024 – 0.169)	0.066 (-0.009 – 0.140)	0.076 (-0.073 – 0.225)	0.916
Playing cards × time	0.092 (0.026 – 0.157)	0.006 (-0.063 – 0.076)	0.196 (0.069 – 0.323)	0.006
Raising pets × time	-0.019 (-0.077 – 0.039)	-0.036 (-0.100 – 0.028)	-0.011 (-0.120 – 0.097)	0.700
Leisure activity score × time	0.018 (0.012 – 0.024)	0.006 (-0.001 – 0.013)	0.026 (0.014 – 0.038)	0.002
Leisure activity frequency				<0.001
T1 × time	Reference	Reference	Reference	
T2 × time	0.119 (0.053 – 0.185)	0.062 (-0.012 – 0.136)	0.134 (0.016 – 0.253)	
T3 × time	0.186 (0.115 – 0.257)	0.066 (-0.011 – 0.143)	0.283 (0.141 – 0.425)	
Leisure activities variety				<0.001
(0-1) × time	Reference	Reference	Reference	
(2-3) × time	0.194 (0.099 – 0.290)	0.001 (-0.125 – 0.126)	0.276 (0.126 – 0.426)	
(4-7) ×time	0.299 (0.198 – 0.399)	0.024 (-0.103 – 0.152)	0.464 (0.292 – 0.635)	

Notes: ADL = activity of daily living, BMI = body mass index.

* A full covariate-adjusted model was used. Full covariates adjustment included sex, age, education, residence, economic status, occupation, exercise, smoking, drinking, diet habit, depression, hypertension, diabetes, stroke/cardiovascular disease, ADL, and BMI.

Table S7. Sensitivity analysis results after restriction to participants with two or more follow-up MMSE assessments

Variables	Overall<br>*β* (95% *CI*)	Married<br>*β* (95% *CI*)	Unmarried<br>*β* (95% *CI*)	*P* for interaction
Watching TV × time	0.338 (0.246 – 0.431)	0.217 (0.093 – 0.342)	0.324 (0.187 – 0.462)	0.264
Gardening × time	0.121 (0.053 – 0.189)	0.041 (-0.031 – 0.113)	0.219 (0.080 – 0.357)	**0.014**
Reading × time	0.109 (0.043 – 0.174)	0.041 (-0.024 – 0.106)	0.116 (-0.026 – 0.258)	0.327
Housework × time	0.028 (-0.052 – 0.107)	-0.035 (-0.121 – 0.050)	0.118 (-0.027 – 0.262)	0.059
Social activities × time	0.091 (0.020 – 0.162)	0.069 (-0.005 – 0.143)	0.058 (-0.090 – 0.206)	0.880
Playing cards × time	0.077 (0.012 – 0.141)	-0.003 (-0.072 – 0.066)	0.179 (0.052 – 0.305)	**0.007**
Raising pets × time	-0.017 (-0.076 – 0.042)	-0.035 (-0.097 – 0.028)	-0.008 (-0.116 – 0.100)	0.653
Leisure activity score × time	0.017 (0.011 – 0.023)	0.005 (-0.001 – 0.012)	0.025 (0.013 – 0.037)	**0.003**
Leisure activity frequency				**<0.001**
T1 × time	Reference	Reference	Reference	
T2 × time	0.120 (0.055 – 0.185)	0.073 (-0.001 – 0.147)	0.130 (0.012 – 0.249)	
T3 × time	0.171 (0.100 – 0.241)	0.065 (-0.011 – 0.142)	0.254 (0.113 – 0.396)	
Leisure activities variety				**<0.001**
(0-1) × time	Reference	Reference	Reference	
(2-3) × time	0.190 (0.093 – 0.288)	0.023 (-0.102 – 0.149)	0.260 (0.104 – 0.416)	
(4-7) ×time	0.289 (0.186 – 0.391)	0.047 (-0.081 – 0.175)	0.433 (0.257 – 0.609)	

Notes: ADL = activity of daily living, BMI = body mass index.

[*] A full covariate-adjusted model was used. Full covariates adjustment included sex, age, education, residence, economic status, occupation, exercise, smoking, drinking, diet habit, depression, hypertension, diabetes, stroke/cardiovascular disease, ADL, and BMI.

Table S8. Sensitivity analysis results after considering subjective social isolation as an independent covariate

Variables	Overall<br>β (95% CI)	Married<br>β (95% CI)	Unmarried<br>β (95% CI)	P for interaction
Watching TV × time	**0.402 (0.309 – 0.495)**	**0.207 (0.087 – 0.326)**	**0.389 (0.227 – 0.551)**	0.109
Gardening × time	**0.178 (0.103 – 0.254)**	0.053 (-0.020 – 0.126)	**0.3 (0.125 – 0.476)**	**0.005**
Reading × time	**0.192 (0.122 – 0.261)**	0.051 (-0.015 – 0.117)	**0.261 (0.086 – 0.436)**	**0.020**
Housework × time	**0.161 (0.080 – 0.241)**	-0.01 (-0.093 – 0.074)	**0.345 (0.186 – 0.504)**	**<0.001**
Social activities × time	**0.151 (0.072 – 0.230)**	**0.085 (0.010 – 0.160)**	0.141 (-0.047 – 0.330)	0.637
Playing cards × time	**0.133 (0.061 – 0.205)**	0.03 (-0.040 – 0.101)	**0.220 (0.060 – 0.381)**	**0.019**
Raising pets × time	0.002 (-0.061 – 0.065)	-0.024 (-0.087 – 0.039)	-0.008 (-0.143 – 0.128)	0.747
Leisure activity score × time	**0.029 (0.023 – 0.036)**	**0.009 (0.002 – 0.016)**	**0.042 (0.028 – 0.057)**	**<0.001**
Leisure activity frequency				**<0.001**
T1 × time	Reference	Reference	Reference	
T2 × time	**0.175 (0.103 – 0.246)**	**0.083 (0.009 – 0.158)**	**0.191 (0.044 – 0.337)**	
T3 × time	**0.286 (0.208 – 0.363)**	**0.099 (0.022 – 0.176)**	**0.413 (0.233 – 0.594)**	
Leisure activities variety				**<0.001**
(0-1) × time	Reference	Reference	Reference	
(2-3) × time	**0.347 (0.250 – 0.444)**	0.021 (-0.098 – 0.140)	**0.490 (0.320 – 0.660)**	
(4-7) ×time	**0.500 (0.397 – 0.604)**	0.077 (-0.045 – 0.198)	**0.698 (0.496 – 0.901)**	

Notes: ADL = activity of daily living, BMI = body mass index.

* A full covariate-adjusted model was used. Full covariates adjustment included sex, age, education, residence, economic status, occupation, exercise, smoking, drinking, diet habit, depression, hypertension, diabetes, stroke/cardiovascular disease, subjective social isolation, ADL, and BMI.

Bold indicates significant results.

Table S9. Age and marital status-specific leisure activity and cognitive decline

Variables	65-74			75+		
	Married	Unmarried	P for interaction	Married	Unmarried	P for interaction
	β (95% CI)	β (95% CI)		β (95% CI)	β (95% CI)	
Watching TV × time	-0.004 (-0.128 – 0.120)	0.014 (-0.195 – 0.223)	0.827	0.247 (0.015 – 0.478)	0.345 (0.147 – 0.543)	0.577
Gardening × time	-0.024 (-0.086 – 0.038)	0.029 (-0.120 – 0.178)	0.441	0.175 (-0.000 – 0.350)	0.384 (0.145 – 0.622)	0.184
Reading × time	0.002 (-0.053 – 0.058)	0.02 (-0.130 – 0.171)	0.846	0.034 (-0.131 – 0.199)	0.323 (0.086 – 0.560)	0.057
Housework × time	-0.039 (-0.119 – 0.041)	-0.027 (-0.281 – 0.227)	0.961	-0.100 (-0.275 – 0.075)	0.268 (0.077 – 0.459)	**0.009**
Social activities × time	0.001 (-0.062 – 0.065)	-0.067 (-0.227 – 0.092)	0.321	0.194 (0.009 – 0.380)	0.181 (-0.073 – 0.436)	0.935
Playing cards × time	-0.008 (-0.066 – 0.051)	0.025 (-0.111 – 0.161)	0.615	0.053 (-0.122 – 0.228)	0.229 (0.012 – 0.447)	0.252
Raising pets × time	-0.033 (-0.086 – 0.020)	0.002 (-0.121 – 0.125)	0.464	-0.097 (-0.254 – 0.060)	-0.072 (-0.251 – 0.107)	0.817
Leisure activity score × time	-0.004 (-0.010 – 0.002)	0 (-0.015 – 0.015)	0.602	0.012 (-0.004 – 0.029)	0.039 (0.021 – 0.058)	**0.043**
Leisure activity frequency			**0.014**			**<0.001**
T1 × time	Reference	Reference		Reference	Reference	
T2 × time	-0.021 (-0.088 – 0.046)	-0.008 (-0.152 – 0.136)		0.117 (-0.055 – 0.289)	0.153 (-0.037 – 0.343)	
T3 × time	-0.035 (-0.102 – 0.031)	0.008 (-0.146 – 0.162)		0.130 (-0.061 – 0.322)	0.427 (0.169 – 0.684)	
Leisure activities variety			**0.002**			**<0.001**
(0-1) × time	Reference	Reference		Reference	Reference	
(2-3) × time	-0.051 (-0.175 – 0.073)	0.124 (-0.163 – 0.411)		-0.065 (-0.293 – 0.163)	0.402 (0.199 – 0.604)	
(4-7) ×time	-0.054 (-0.179 – 0.071)	0.105 (-0.191 – 0.400)		-0.004 (-0.250 – 0.242)	0.670 (0.409 – 0.931)	

Notes: ADL = activity of daily living, BMI = body mass index. Bold indicates significant results.
[*] Full covariates adjustment included sex, age, education, residence, economic status, occupation, exercise, smoking, drinking, diet habit, depression, hypertension, diabetes, stroke/cardiovascular disease, ADL, and BMI.

Table S10. Sex and marital status-specific leisure activity and cognitive decline

Variables	Man			Female		
	Married	Unmarried	P for interaction	Married	Unmarried	P for interaction
	β (95% CI)	β (95% CI)		β (95% CI)	β (95% CI)	
Watching TV × time	0.178 (0.006 – 0.349)	0.158 (-0.131 – 0.446)	0.866	0.209 (0.031 – 0.387)	0.432 (0.236 – 0.629)	0.179
Gardening × time	0.04 (-0.052 – 0.132)	0.129 (-0.112 – 0.370)	0.374	0.071 (-0.057 – 0.200)	**0.377 (0.152 – 0.602)**	**0.038**
Reading × time	0.011 (-0.070 – 0.091)	-0.036 (-0.258 – 0.186)	0.565	0.093 (-0.046 – 0.232)	0.432 (0.157 – 0.706)	0.053
Housework × time	-0.018 (-0.111 – 0.076)	**0.336 (0.131 – 0.542)**	**0.001**	0.213 (-0.037 – 0.464)	0.412 (0.197 – 0.628)	0.414
Social activities × time	0.060 (-0.034 – 0.153)	-0.062 (-0.310 – 0.186)	0.317	0.126 (-0.011 – 0.262)	0.236 (-0.012 – 0.485)	0.597
Playing cards × time	0.052 (-0.034 – 0.139)	0.198 (-0.035 – 0.431)	0.174	-0.03 (-0.161 – 0.102)	0.193 (-0.019 – 0.405)	0.083
Raising pets × time	0.025 (-0.056 – 0.107)	0.053 (-0.145 – 0.252)	0.853	-0.092 (-0.200 – 0.016)	-0.011 (-0.181 – 0.159)	0.291
Leisure activity score × time	0.008 (-0.000 – 0.016)	0.024 (0.003 – 0.046)	0.136	0.011 (-0.002 – 0.024)	**0.049 (0.031 – 0.067)**	**0.003**
Leisure activity frequency			**<0.001**			**<0.001**
T1 × time	Reference	Reference		Reference	Reference	
T2 × time	0.021 (-0.077 – 0.119)	0.032 (-0.203 – 0.267)		0.185 (0.060 – 0.310)	**0.251 (0.068 – 0.434)**	
T3 × time	0.057 (-0.040 – 0.153)	0.197 (-0.067 – 0.461)		0.164 (0.026 – 0.303)	**0.488 (0.248 – 0.729)**	
Leisure activities variety			**<0.001**			**<0.001**
(0-1) × time	Reference	Reference		Reference	Reference	
(2-3) × time	0.035 (-0.110 – 0.180)	**0.368 (0.095 – 0.642)**		0.017 (-0.210 – 0.245)	**0.555 (0.342 – 0.768)**	
(4-7) ×time	0.081 (-0.066 – 0.227)	**0.469 (0.165 – 0.772)**		0.073 (-0.164 – 0.309)	**0.785 (0.521 – 1.050)**	

Notes: ADL = activity of daily living, BMI = body mass index. Bold indicates significant results.

* Full covariates adjustment included sex, age, education, residence, economic status, occupation, exercise, smoking, drinking, diet habit, depression, hypertension, diabetes, stroke/cardiovascular disease, ADL, and BMI.

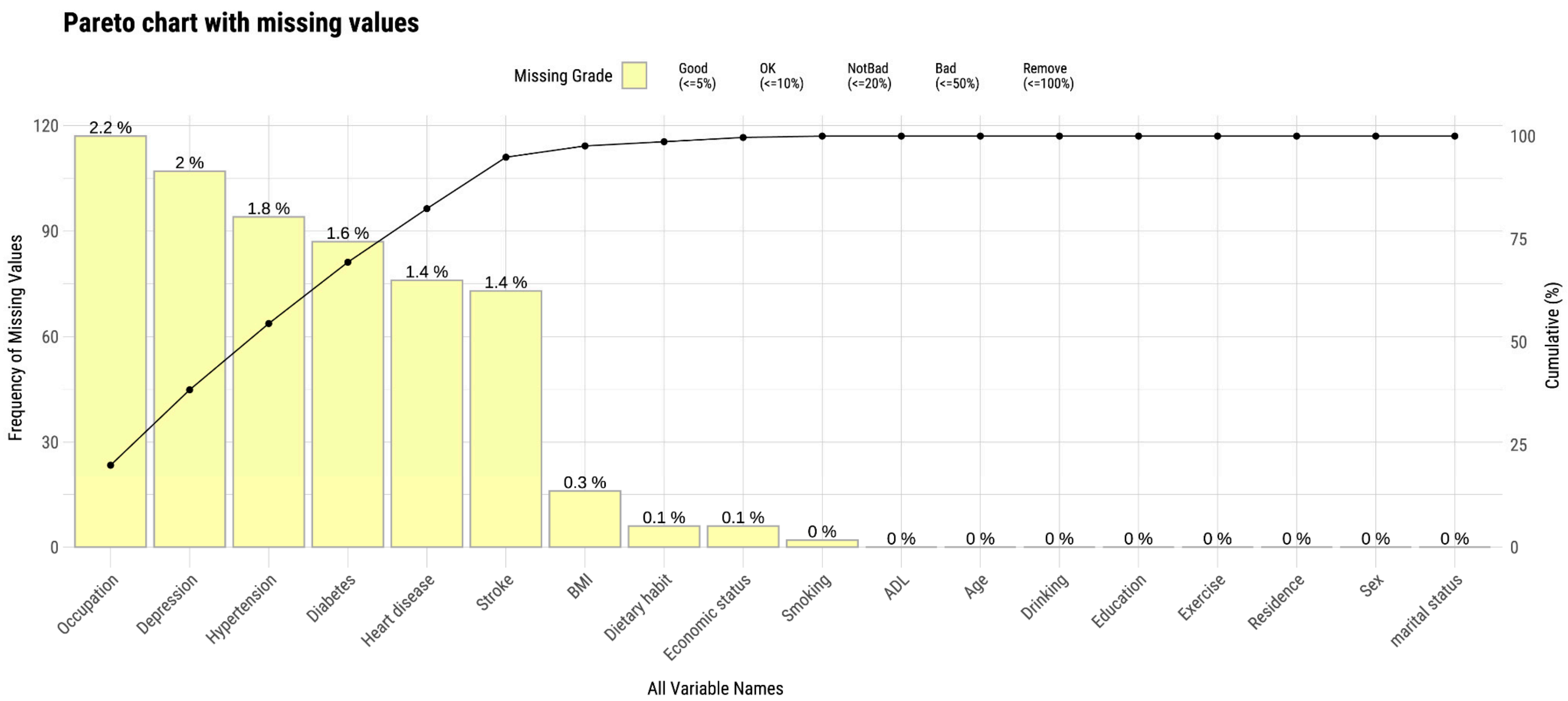

Figure S1. Proportion of missing data at baseline for covariates
